# The impact of threat of shock-induced anxiety on the neural substrates of memory encoding and retrieval

**DOI:** 10.1093/scan/nsz080

**Published:** 2019-10-31

**Authors:** Michele Garibbo, Jessica Aylward, Oliver J Robinson

**Affiliations:** Institute of Cognitive Neuroscience, University College London, WC1N 3AZ, London, United Kingdom

**Keywords:** anxiety, memory, fMRI, threat of shock, ACC

## Abstract

Dysfunctional memory processes are widely reported in anxiety disorders, but the underlying neurocognitive mechanisms are unclear. Recent work shows that the impact of anxiety on memory depends on the context and memory modality. For instance, threat of shock, a translational within-subject anxiety induction, has been shown to impair the encoding of facial stimuli, while improving spatial working memory (WM) accuracy. The present study aimed to delineate the neural circuitry regulating these opposing behavioural effects. Thirty-three healthy volunteers performed the previously assessed facial recognition and a spatial WM tasks inside an fMRI scanner, under alternating within-subject conditions of threat or safe from shock across encoding and retrieval. Facial recognition impairments were replicated when threat was selectively induced at encoding. Neuroimaging results suggest that this effect was driven by increased competition for attentional resources within the anterior cingulate cortex, in which activation correlated positively with stress levels. The impact of threat on spatial WM performance did not, however, replicate in the fMRI environment. Nevertheless, state-dependent hippocampal activation was observed in both tasks. These findings suggest a neurocognitive mechanism by which anxiety impairs facial recognition as well as a state-dependent hippocampal activation pattern, which may putatively underline retrieval of negative experiences in anxiety.

## Introduction

Anxiety disorders are the most common mental health problem worldwide with an averaged estimated lifetime prevalence of 16% (Kessler *et al.*, 2009). In addition to the direct burden of the anxious state, anxiety disorders are associated with additional cognitive symptoms. For instance, memory alterations are commonly observed in anxiety disorders (Eysenck and Calvo, 1992; Airaksinen *et al.*, 2005; Mantella *et al.*, 2007; Zlomuzica *et al.*, 2014; Moran, 2016). Recent experimental studies have attempted to understand memory–anxiety interactions during specific memory stages (e.g. encoding *vs* retrieval) and/or modalities (e.g. facial recognition *vs* spatial memory) (Vytal *et al.*, 2012; Robinson *et al.*, 2013; Vytal *et al.*, 2013; Moran, 2016). Bolton and Robinson (2017), in particular, demonstrated that threat of shock (ToS)-induced anxiety (i) impaired encoding of facial stimuli, but (ii) improved spatial working memory (WM) in a state-dependent manner. In this paper, we attempt to replicate these findings, while exploring the underlying neurobiological activity using functional magnetic resonance imaging.

Face recognition abnormalities occur in both anxiety disorders (e.g. Surcinelli *et al.*, 2006; Dickie *et al.*, 2008; Jarros *et al.*, 2012) and induced anxiety (Attwood *et al.*, 2013; Bolton and Robinson, 2017). This may be because face encoding is affected by attentional allocation (Brown *et al.*, 1997; Palermo and Rhodes, 2002; Jackson and Raymond, 2006) such that reduced attentional allocation to facial stimuli leads to reduced information processing across behavioural and neural measurements (see Pessoa *et al.*, 2002). Anxiety, moreover, promotes stimulus-driven bottom-up attention at the expense of top-down sustained attention (Eysenck *et al.*, 2007; Bishop, 2009) (see also Corbetta and Shulman, 2002). Consequently, anxiety-related attentional resource allocation may lead to reduced face processing and hence recognition impairments when anxiety is selectively present at encoding.

At the neural level, faces are processed by a `core’ of structures required for forming holistic facial representations (i.e. fusiform gyrus, inferior occipital gyrus and superior temporal sulcus) (Kanwisher *et al.*, 1997), but critically, with additional involvement of `domain-general’ areas required for top-down attentional control including anterior cingulate cortex (ACC) and medial prefrontal cortices (mPFC) (Duncan and Owen, 2000; Hopfinger *et al.*, 2000; Palermo and Rhodes, 2007). These attention-related areas are, however, also implicated in anxiety processing and attention towards threat-related stimuli (Robinson *et al.*, 2016). As such anxiety induction might impair facial recognition (Bolton and Robinson, 2017) through increased neural resource allocation in these regions. This may therefore constitute a neural instantiation of the attentional resource allocation account described above.

Spatial WM alterations are also seen across anxiety disorders (e.g. van der Wee *et al.*, 2003; Boldrini *et al.*, 2005) and induced anxiety (e.g. Shackman *et al.*, 2006; Robinson *et al.*, 2013; Vytal *et al.*, 2013). While some studies demonstrate anxiety-related impairments, a recent study employing ToS with healthy volunteers found that when anxiety is induced during both encoding and retrieval, a state-dependent improvement in spatial memory is seen (Bolton and Robinson, 2017). Such a state-dependent process may underline excessive retrieval mechanisms of negative experiences in post-traumatic stress disorder (PTSD) and obsessive-compulsive disorder (OCD). For instance, a traumatic experience may become associated with the state of anxiety, such that when anxiety is reencountered, it facilitates recall of the traumatic experience. At the neural level, the hippocampus may be involved in binding item and contextual information together at encoding and reactivating the specific association at recall, facilitating retrieval processing (Diana *et al.*, 2007; Montaldi and Mayes, 2010; Eichenbaum *et al.*, 2012; Martin *et al.*, 2016). Consequently, when anxiety is reinstated at the retrieval of information encoded in the same state, hippocampal-related reactivation of the item-context association may facilitate recall.

The present study therefore aimed to (i) replicate the behavioural findings of Bolton and Robinson (2017) and (ii) extend this to identify the neural bases of these memory effects. We hypothesised that at encoding, ToS would impair face recognition performance and this effect will be driven by functional changes in structures involved in top-down attention (e.g. ACC/mPFC). We also hypothesised that anxiety would trigger a state-dependent memory improvement in spatial memory, and this behavioural effect would be reflected by increased hippocampal activation at retrieval when the emotional state matched that of encoding.

## Methods

### Participants

The final sample of 32 and 33 participants for the face recognition and spatial span task, respectively, was determined according to the effect size of the spatial task of Bolton and Robinson (2017) (Cohen’s *d* = 0.48). *N* = 33 is sufficient to achieve 80% at alpha = 0.05, for a one-tailed *t*-test on the behavioural effect. The use of a one-tailed *t*-test was based on a clear prediction of the direction of the behavioural effect, provided by the previous study (i.e. Bolton and Robinson, 2017). The power analysis exclusively applies to the behavioural effects. Given the replicative nature of the present investigation, this analysis was carried out to make sure that any potential failure to replicate the previously observed behavioural effect could not be attributed to statistical testing not having enough power.

The exclusion criteria were (i) general Functional magnetic resonance imaging (fMRI) exclusions, (ii) general ill health, (iii) family or personal history of psychiatric disorders, including drug or alcohol abuse and (iv) use of illicit drugs or medications within the previous 3 months. All criteria were assessed through a phone screening interview.

### Measures

The order of the facial recognition and spatial span memory tasks was randomly counterbalanced across participants. TOS was administered following a standardised shock work-up procedure using a Digitimer DS7 (see Robinson *et al.*, 2012, 2016).

Both tasks were divided into four blocks and carried out under safe and threat conditions. Each block was one of the four possible combinations of threat and safe conditions at encoding and retrieval. The presentation order of the four blocks was randomly counterbalanced across participants in each task. A 30 s fixation preceded and followed each task to provide additional baseline for fMRI contrasts. At the end of each task, retrospective ratings of self-reported stress were collected by voice via a provided microphone, on a scale from 1 (i.e. not stressed at all) to 10 (i.e. extremely stressed). Additionally, a computerised version of the State-Trait Anxiety Inventory (STAI) was administered to each participant before completing the tasks. Finally, a short 4 min practice version was completed twice: once outside of the scanner (i.e. on a computer laptop) and once inside the scanner.

### Facial recognition

**Fig. 1:**
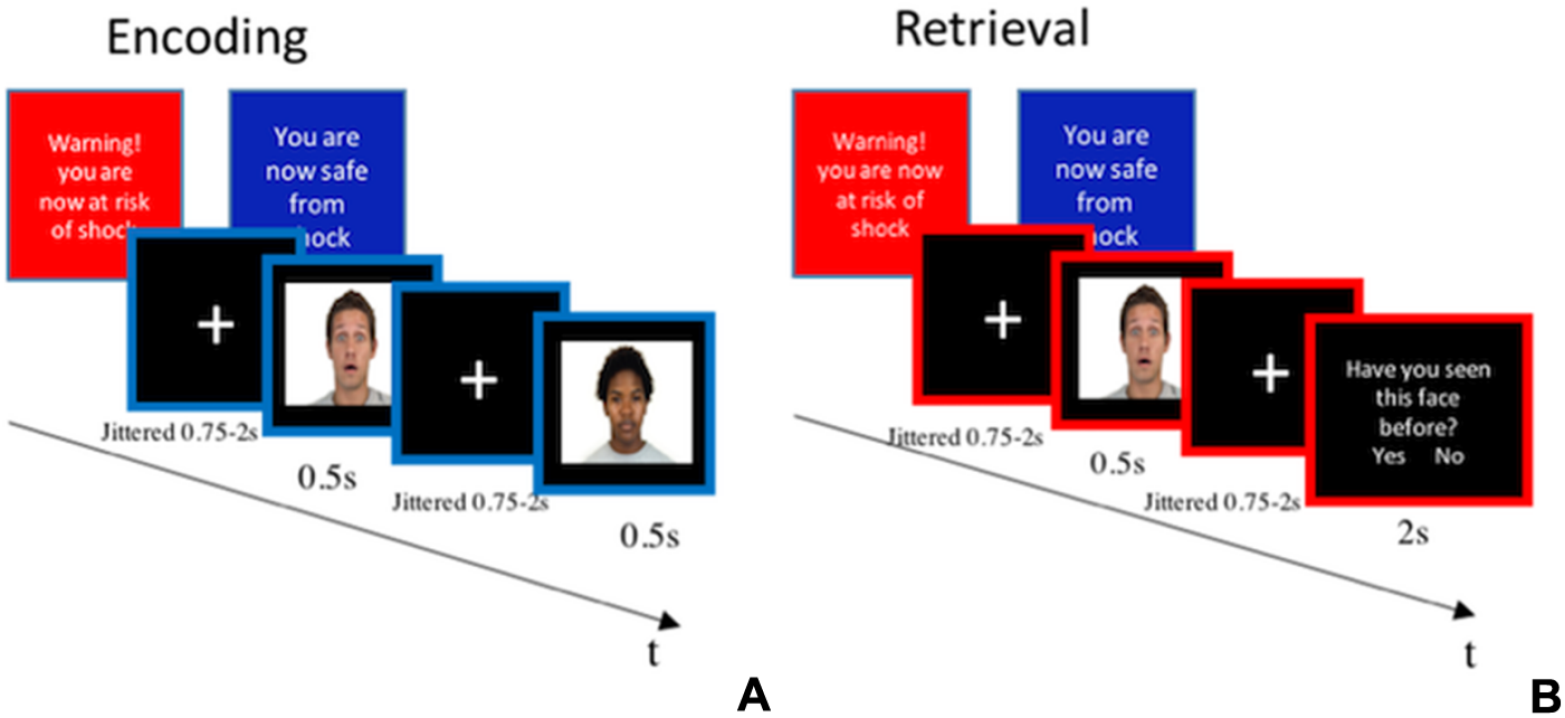
(A) Encoding and (B) retrieval phase of the facial recognition memory task.

Each block consisted of a different set of 36 facial stimuli (i.e. 144 overall) selected from the Chicago Face Database (Ma *et al.*, 2015). Each set comprised an equal number of male and female as well as happy, sad and neutral faces. During encoding, 18 facial stimuli were presented to participants. The stimuli were displayed one at the time, and they were separated by a fixation interval (i.e. ISI) (Figure 1A). The encoding phase was followed by a fixation interval randomly jittered between 9 and 12 s, which preceded the retrieval phase. At retrieval, 36 faces were presented individually, half of which consisted of the faces seen at encoding, while the other half were completely new ones. After each stimulus, participants were asked to indicate whether they had seen the face in the set before (i.e. during encoding). In order to respond, participants had to press either of two buttons, representing `Yes’ (i.e. I have seen the face before) or `No’ (i.e. I have not seen it before) within an interval of 2 s (Figure 1B). In case this time expired, the response was considered as incorrect. The two buttons respectively corresponding with `Yes’ and `No’ responses were counterbalanced across participants. The dependent variable was the proportion of faces correctly identified as seen before (`Yes’ response) plus the proportion of those correctly identified as not seen before (`No’ response). This task was ~15 min long. The practice version of this task had 1 block only of 6 pictures at encoding and 12 at retrieval, without ToS. Different facial stimuli were employed in the practice compared to the main task.

**Fig. 2:**
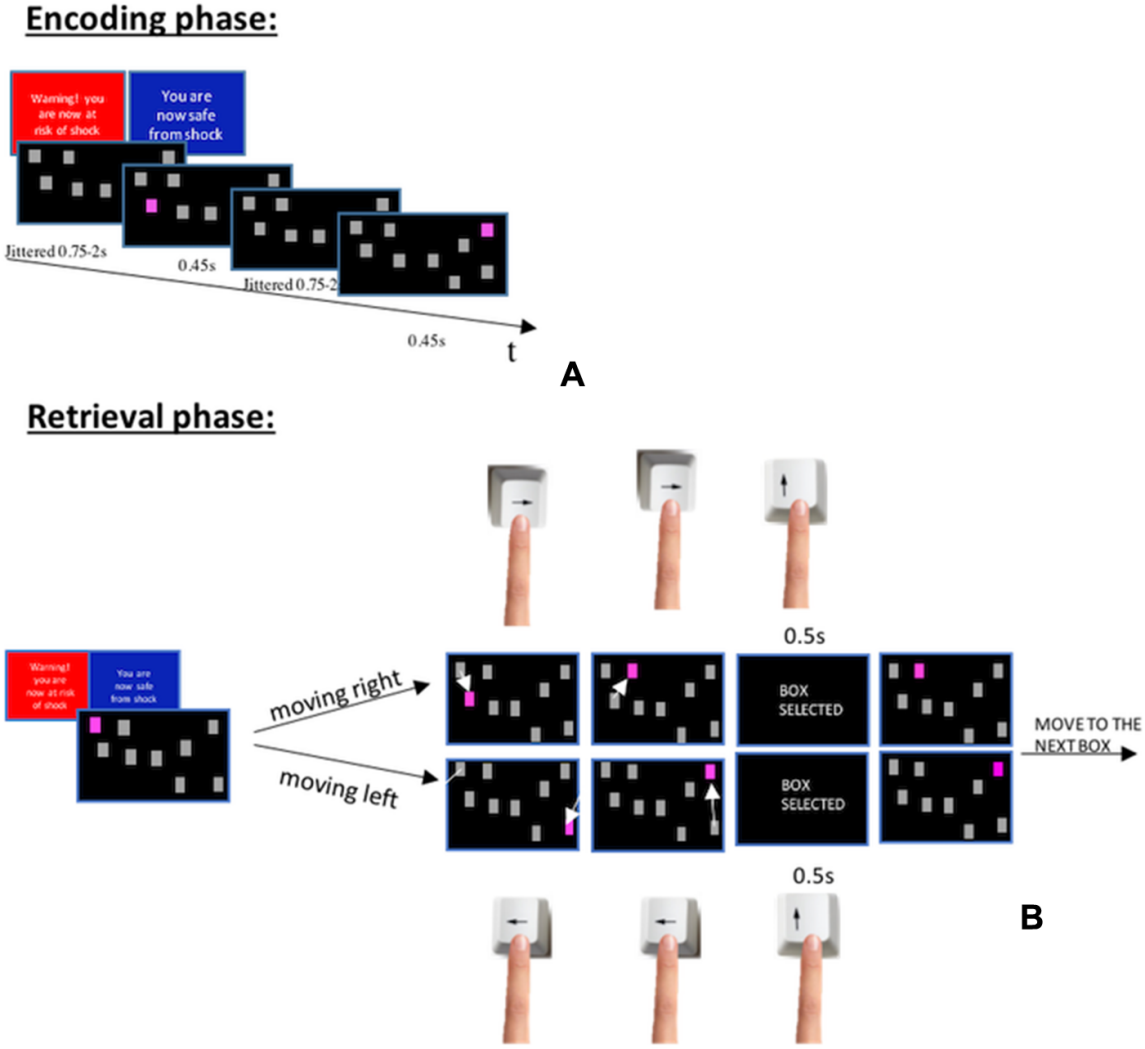
(A) Encoding and (B) retrieval phase of the spatial span task.

### Spatial span (to assess visuospatial WM)

The spatial span task included four blocks with eight trials each. In all trials, nine grey boxes were presented on a black screen. In the encoding phase of the first trial, two of the nine displayed boxes sequentially lit up in a pseudorandom order (Figure 2A). The encoding phase was followed by a fixation interval randomly jittered between 3 and 5 s. After this fixation interval, the retrieval phase presented all the nine boxes in grey colour and required participants to recall and select the two boxes in the exact order in which they had lit up during encoding (Figure 2B). For the selection of each box, participants had 5.5 s (e.g. 2 boxes = 11 s overall). In case the 5.5 s expired, the response for that box was considered incorrect. The following seven trials within each block followed the same procedure but with successively increased numbers of boxes lighting up (i.e. three boxes lighting up at the second trial, four boxes at the third trial all the way to nine boxes).

A fixation interval separated each trial: the length of this fixation interval was equal to the maximum retrieval time for that trial minus the reaction time (RT) of the responses (e.g. 11 s—RT for the first trial), so that the length of the task was standardised across participants. The location of the boxes on the screen changed across the four blocks. In each trial, every box retrieved in the correct order was recorded as one point. Therefore, one or multiple errors on a sequence of boxes to be recalled did not render the entire trial as an error. This task lasted for ~34 min. The practice version, which consisted of three trials only (i.e. from two to four boxes to be remembered) and six instead of nine boxes, were displayed on the screen. The procedure was the same as the main task but without ToS.

**Fig. 3:**
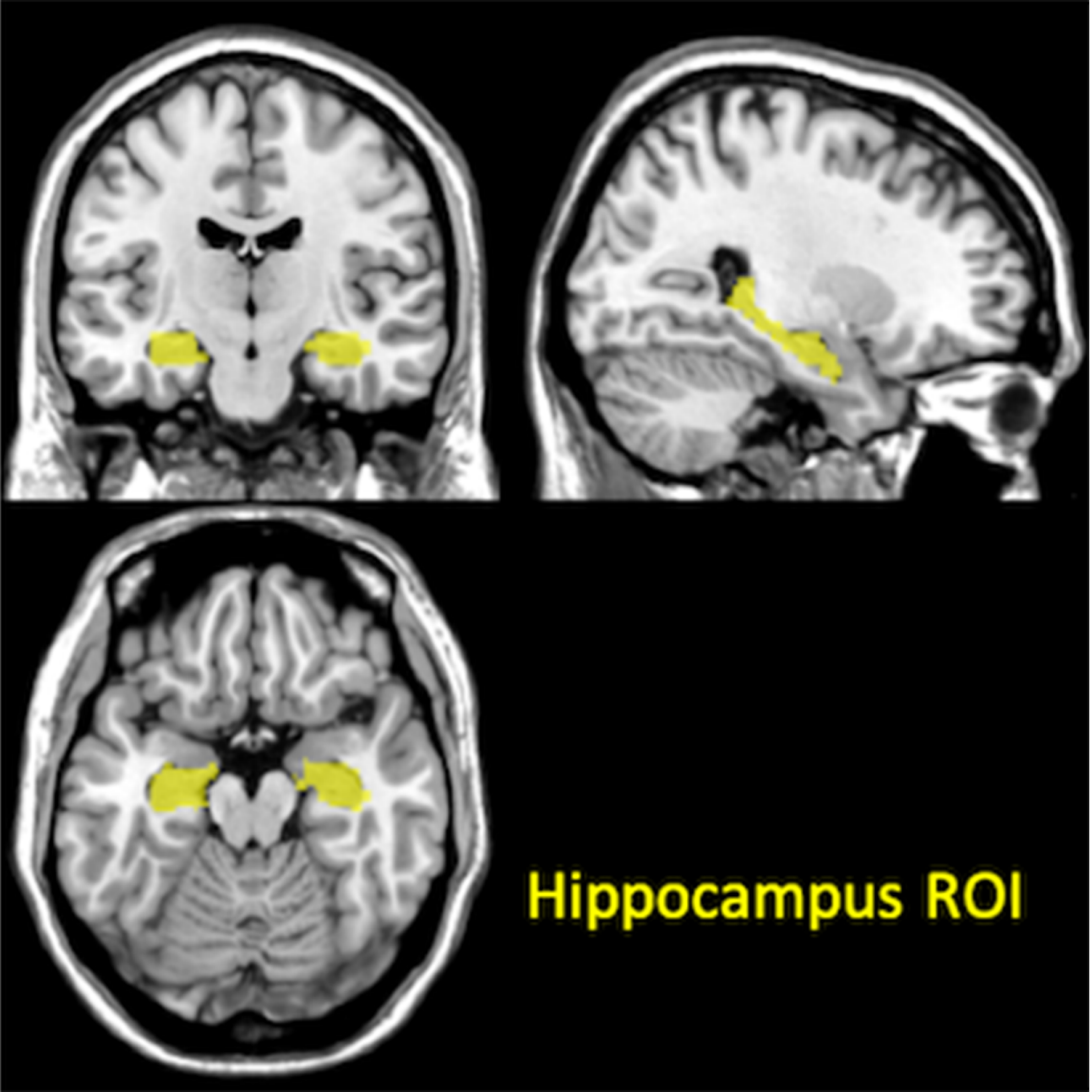
Anatomical location of the employed ROI for the fMRI analysis, the yellow region represents the hippocampus.

The retrieval phase was the only part of the task to differ slightly from Bolton and Robinson (2017) spatial span task, since it had to be adapted to the fMRI environment. In Bolton and Robinson (2017) study, participants had unlimited time to recall each spatial configuration and instead of having to retrieve boxes by moving around the screen with left and right buttons (Figure 2B) they had a button for each corresponding box (i.e. nine buttons in total).

### Pilot study

Prior to the fMRI study, an initial pilot study was conducted involving 20 additional participants, who did not overlap with the participant pool of the main analysis. This study consisted of the same exact measures, tasks and procedures as the fMRI task but was completed outside of the scanner. Both tasks were completed on a computer laptop, and the shock was delivered on the right wrist instead of the left ankle.

### Experimental design and statistical analysis

The analysis consisted of a 2 × 2 within-subject factorial Analysis of variance (ANOVA) for both the facial recognition and spatial span task (i.e. safe-encoding/safe-retrieval, safe-encoding/threat-retrieval, threat-encoding/safe-retrieval and threat-encoding/threat-retrieval). The primary dependent variable for both tasks was the proportion of correct responses.

**Fig. 4:**
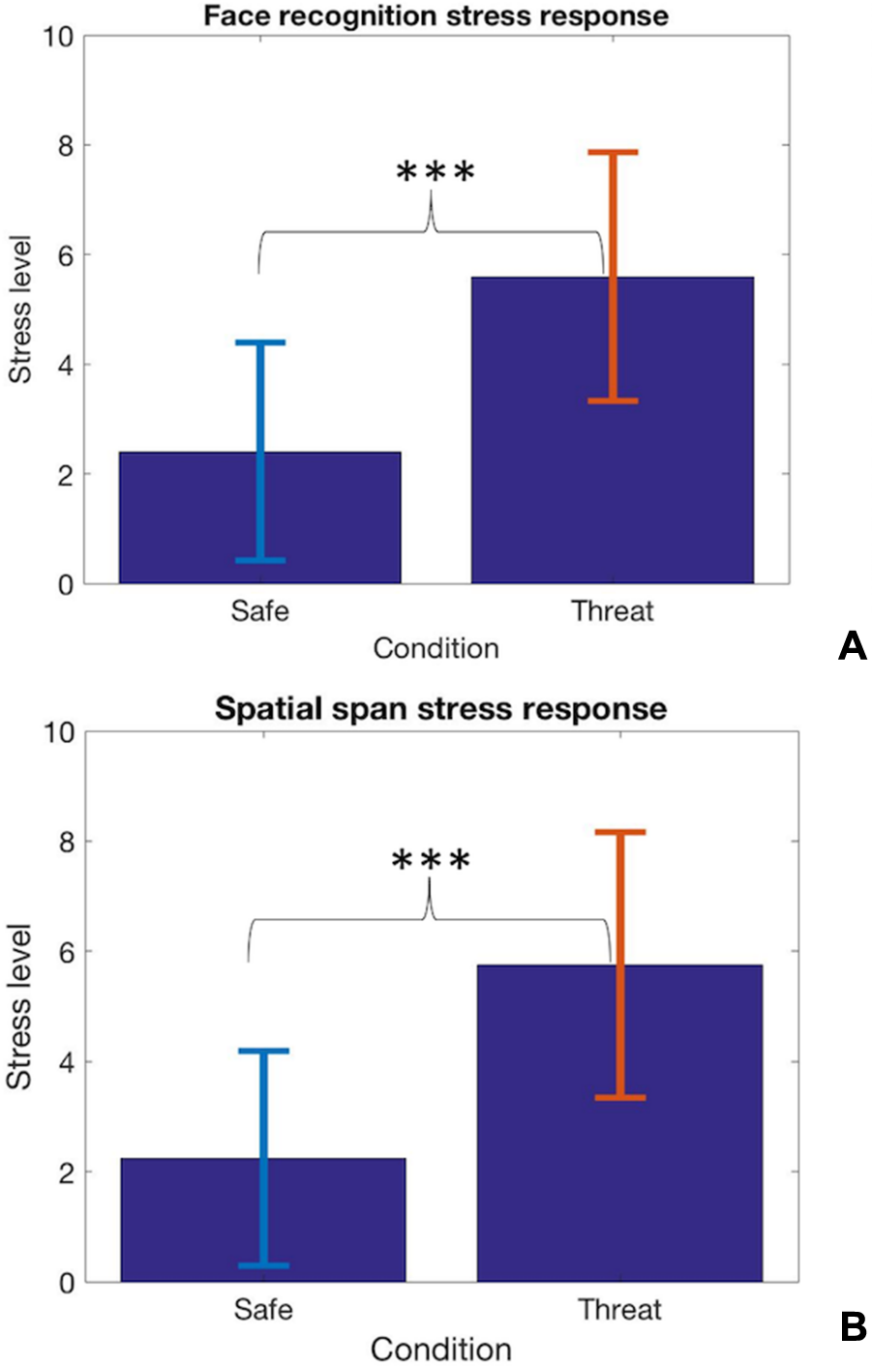
Bar charts representing the difference between the safe and the threat condition in self-reported measure of stress (^***^P < 0.001); (A) facial recognition task. (B) Spatial span task.

**Fig. 5:**
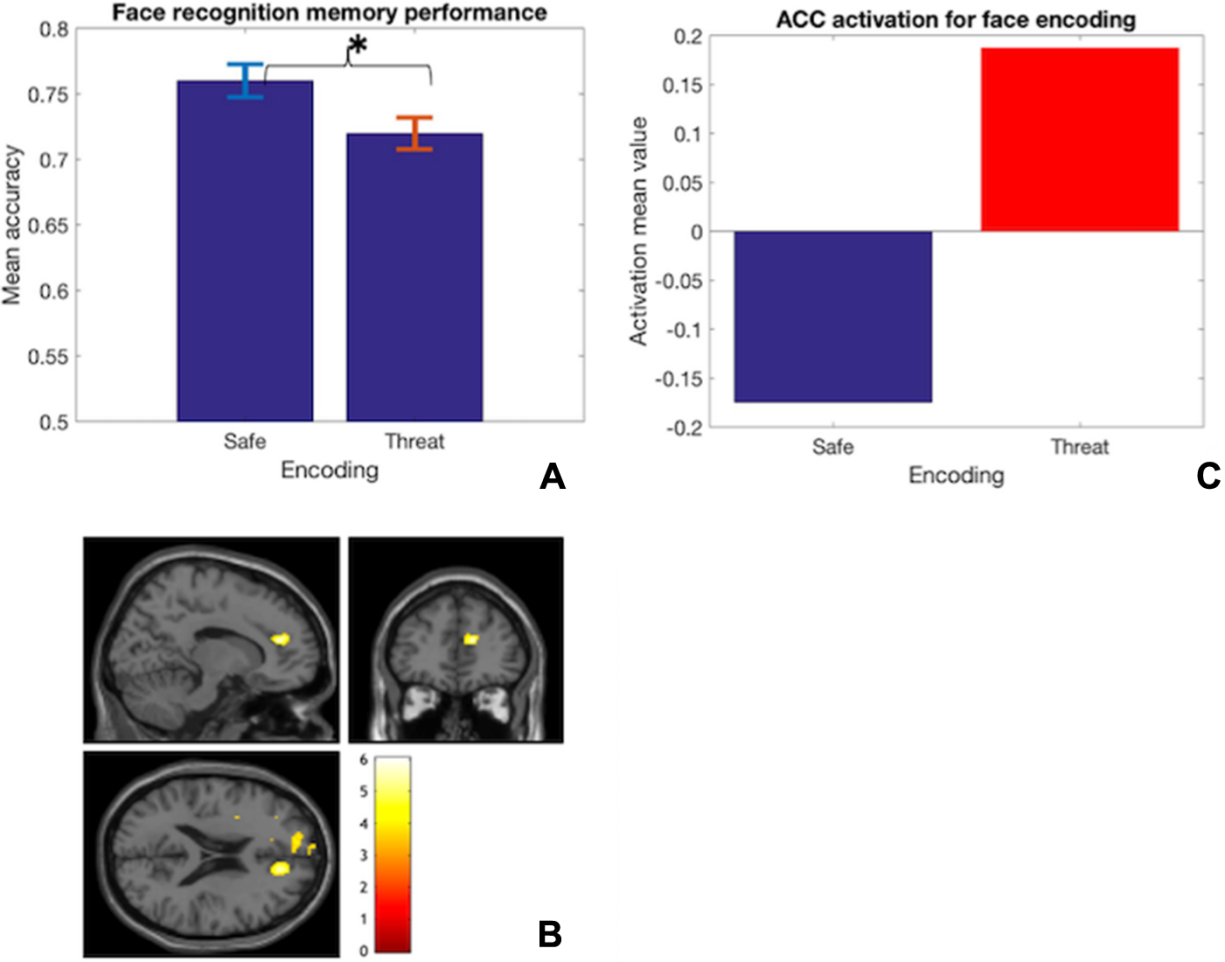
(A) Impaired face recognition accuracy performance when threat is induced at encoding (^*^P < 0.05). (B) Increased ACC activation during threat at encoding relative to the safe condition. (C) ACC mean beta values for safe and threat encoding of facial stimuli.

**Fig. 6:**
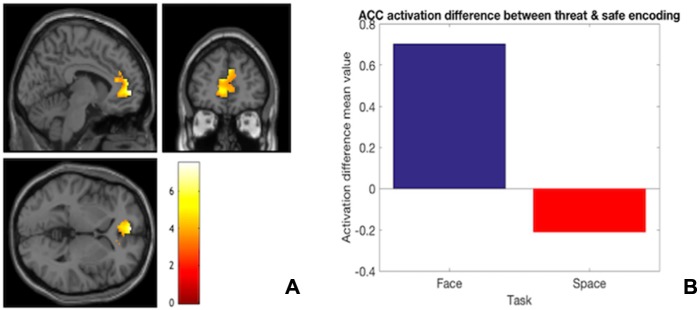
(A) ACC activation difference between the facial and spatial task during threat encoding relative to safe encoding. This shows that anxiety-related ACC activation at encoding may relate to facial rather than spatial stimuli. (B) ACC means beta values for the spatial and facial task during threat encoding relative to safe encoding, showing a higher ACC activation for faces over spatial configurations.

### Functional magnetic resonance imaging

A Siemens Avanto 1.5T MRI scanner was employed to acquire whole-brain gradient-echo T2*-weighted images. A 32-channel head coil was used in combination with foam cushions (to restrict head movement), each volume comprised of 40 slices with a slice gap and slice thickness of 2 mm and a 50% distance factor. A 30° tilted sequence was employed for every echo plan imaging (EPI) sequence. Two EPIs were acquired with identical parameters, one for each task. Echo time was 50 ms with 87.5 ms repetition time for each slice (acquired in a sequential fashion) and 2 × 2 mm in-plane resolution (i.e. repetition time TR = 3.5 s). Two field maps were acquired for every subject with matching parameters to the EPI scan, one at the end of each task in order to allow for correction of distortions in the EPI. A 5 min magnetization-prepared rapid gradient-echo T1-weighted was also acquired for each subject but not used in the analyses. Each participant attended one scanning session, which lasted for 1 h and 10 min approximately. Statistical Parametric Mapping (SPM 12, v6906; Wellcome Trust Centre for Neuroimaging, London, https://www.fil.ion.ucl.ac.uk/spm/) was employed to pre-process and analyse EPI data of the two tasks in Matlab R2017a.

### EPI pre-processing

During data pre-processing of both task EPI, the first six TRs were discarded in order to account for magnetic stabilisation. Realignment and unwarping were conducted employing voxel displacement maps, computed from each EPI field maps. Successively, normalisation to a standardised space (Montreal Neurological Institute template) was carried out on realigned and unwarped data with a spatial resolution of (2 × 2 × 2 mm). Finally, normalised data were smoothed employing an 8 mm full width at half-maximum Gaussian kernel. A standard high pass filter of 128 s was employed.

### fMRI analysis

Across all EPI analysis, a generalised linear model was employed, where each regressor of interest was convolved with SPM canonical synthetic hemodynamic response function, time-locked to the beginning of each block. The regressors of no interest included six movement-correction parameters and three parameters, respectively, controlling for the time at which a shock was delivered, the presentation time of the safe/threat warnings and the message indicating the end of a block. These regressors of no interest were included in the models describing both tasks.

In the facial recognition task, pre-processed data were modelled as a block design for the duration of the encoding and of the retrieval phase separately for each of four blocks within the task. The spatial task was also modelled as a block design, corresponding with the duration of each of the eight trials within the four blocks of the task (i.e. boxcar design), separating encoding from retrieval. The four possible combinations of memory stage by condition (safe-encoding/safe-retrieval, safe-encoding/threat-retrieval etc.) during activation at encoding and activation at retrieval were modelled in both tasks. Finally, data from the two tasks were pooled together, employing the above-described conditions. This data pooling served to investigate activation differences and similarities between the two tasks. `Baseline’ activation was implicit: it comprised the initial and final as well as within trial/block fixation time.

A random effect analysis (Friston *et al.*, 1999) was employed to estimate changes in blood-oxygen level-dependent signal across all participants for both tasks. Second-level contrasts comprised comparisons across the above-described conditions through one-sample *t*-tests. Random effect analysis was conducted for both whole-brain and region of interest (ROI; see below) analysis. All peak-level and cluster-level analysis are reported with the significance threshold set a *P* < 0.05 family-wise error (FWE) corrected for multiple comparison (indicated as FWE_peak and FWE_cluster, respectively). In the cluster-level analysis, all the cluster-forming thresholds were set at *P* < 0.001, which has been shown to control well for false-positive activity, unlike less conservative primary thresholds (e.g. *P* < 0.05) (Woo *et al.*, 2014). All coordinates are reported according to the Montreal Neurological Institute (MNI) coordinate system.

### ROI analysis

One ROI was specified a priori to conduct a single restricted voxel-wise analysis. This region consisted of the hippocampus due to its involvement in state-dependent memory effects (Diana *et al.*, 2007) (Figure 3).

The bilateral hippocampus anatomical ROI mask was extracted from the Wake Forest University PickAtlas toolbox, employing the automated anatomical labelling template (Tzourio-Mazoyer *et al.*, 2002). ROI analysis is corrected for multiple comparison through small volume correction (SVC) at both peak and cluster levels (indicated as FWE_peak_SVC and FWE_cluster_SVC, respectively). FWE_peak_SVC and FWE_cluster_SVC had the significance threshold set at *P* < 0.05 and the cluster-forming threshold at *P* < 0.001.

## Results

**Fig. 7:**
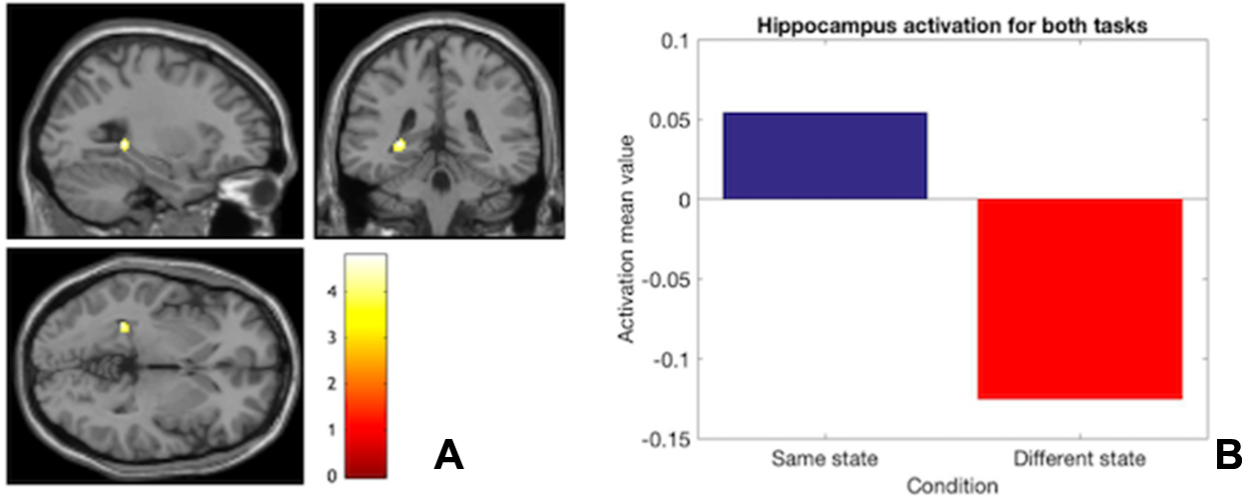
(A) State-dependent ROI hippocampal activation for both tasks pooled together relative to when safe and threat conditions mismatched across the encoding and retrieval stages (B) Hippocampus means beta value for both tasks pooled together.

Overall, face recognition performance was impaired when participants selectively encoded faces under threat. This effect was reflected by increased ACC activation during threat encoding relative to safe. No neural or behavioural changes were observed in relation to the spatial span task during threat. State-dependent, task-independent hippocampal activation was observed when the encoding state was reinstated at retrieval in both tasks, despite no observable behavioural changes. The specifics are reported below and in Figures 4–7 and Tables 1–4.

### Anxiety manipulation

In the spatial span task, participants reported being significantly more stressed during threat [mean (SD) = 5.76(2.4)] than safe conditions [mean (SD) = 2.24(1.95)]; *t*(32) = 8.6; *P* < 0.001, two tailed (Figure 4A). Similarly, participants reported being significantly more stressed in the threat [mean (SD) = 5.59(2.3)] relative to safe conditions [mean (SD) = 2.41(1.98)] during the face recognition memory task; *t*(31) = 9.62; *P* < 0.001, two tailed (Figure 4B).

### Face recognition task

#### Behavioural analysis

The repeated-measures 2 × 2 ANOVA revealed a significant main effect for encoding (*F*(1,31) = 4.4, *P* = 0.045, estimated partial eta square = 0.123), replicating Bolton and Robinson (2017) finding. This effect represented participants achieving better memory performance when encoding information under safe [mean (SD) = 0.76(0.14)], compared to threat, [mean (SD) = 0.72(0.14)] (Figure 5A). There was no significant main effect for retrieval nor was the interaction effect significant (*P* > 0.05).

**Table 1:** Total values or means of demographic variables

	Face recognition	Spatial span
Gender		
Female	18	19
Male	14	14
Age	27.03	27.41
State anxiety	33.22(10.65)	32.51(9.95)
Trait anxiety	35.78.(9.80)	35.13(9.43)

Note*. N* = 32 and 33 for the face recognition and spatial span memory tasks; state and trait anxiety scores are based on the STAI; the standard deviations of these scores are represented within brackets.

#### fMRI analysis

Whole-brain analysis revealed the ACC was significantly more activated at the cluster level during encoding under threat relative to safe encoding (Table 2) (Figure 5C). Across the whole brain, this ACC activation for threat *vs* safe encoding was also nearly significant at peak level (Table 2). The whole-brain analysis was run as an exploratory analysis to investigate the unknown overall neural changes underlying the observed behavioural effect. Successively, beta values of each participants’ activation of the ACC (peak-level xyz coordinates, 12 40 18) for this comparison were extracted and found to positively correlate with self-reported stress levels, based on relative scores, Pearson’s *r*(32) = 0.32; *P* = 0.038 (one tailed—predicted positive relationship). Relative scores were obtained by computing the difference in self-reported stress levels between the threat and safe conditions (e.g. Kaye *et al.*, 2016; Kirlic *et al.*, 2019). The use of a one-tailed test to assess the significance of the correlation was justified based on previous extensive evidence of subjective anxiety and perceived threat positively relating to ACC activation (e.g. Amir *et al.*, 2005; Phan *et al.*, 2006; Straube *et al.*, 2007; Straube *et al.*, 2009).

### Spatial span task

#### Behavioural analysis

There was no significant main effect for encoding or retrieval (*P* > 0.05). Inconsistent with the prior study, the interaction between encoding/retrieval and safe/threat did not reach significance either (*P* > 0.05).

#### fMRI analysis

In line with the behavioural findings, there was no whole-brain significance difference in activation between the safe and the threat conditions at encoding (*P* < 0.05 FWE_peak and FWE_cluster).

### Comparison across tasks

In this section, neural data from the two tasks were included in the same model to directly compare the above ACC effects to the encoding of stimuli (i.e. task differences). Additionally, neural data from the two tasks were pooled together and a state-dependent, task-independent hippocampal activation was investigated across the two tasks (i.e. task consistency).

#### Task differences

ACC whole-brain activation was compared between the two tasks during encoding under threat relative to the safe condition. This analysis revealed that the ACC was significantly more activated during the encoding of facial stimuli in the threat *vs* safe condition relative to spatial stimuli in the same contrast (Table 4, task difference, and Figure 6).

**Table 2:** ACC during threat encoding compared to safe encoding of the facial recognition task

	*k*	*P* (FWE_cluster)	*P* (FWE_peak)	*t*-score	Z-score	Coordinates
Task difference (facial > spatial in threat *vs* safe encoding)						
ACC	144	.014	.09	6.05	4.88	12 40 18

**Table 3:** Behavioural data of both tasks. Values represent accuracy under conditions of safe and threat at encoding and retrieval

Spatial span: *N* = 33				
	SF/SF	SF/TH	TH/SF	TH/TH
	0.57(0.15)	0.57(0.16)	0.58(0.19)	0.55(0.17)
Face recognition = 32				
	0.74(0.15)	0.77(0.14)	0.73(0.13)	0.71(0.14)

Note. Mean (SD) of untransformed data; encoding/retrieval; SF = safe; TH = threat.

**Table 4:** Comparison of ACC activation between the two tasks and state-dependent hippocampal ROI activation of the two tasks pooled together

	*k*	*P* (FWE_cluster)	*P* (FWE_peak)	*t*-score	Z-score	Coordinates
Task difference (facial > spatial in threat *vs* safe encoding)						
ACC	762	<0.001	<0.001	7.52	5.59	−4 52 12
Task consistency (facial and spatial in condition matching *vs* mismatching across memory stages)						
Hippocampus (ROI)	32	0.022	0.018	4.79	4.09	−30-40 0

#### Task consistency

When the condition was matched across encoding and retrieval for neural data pooled from the two task, ROI hippocampal activation was significantly higher compared to when it mismatched across memory stages (i.e. safe/safe and threat/threat *vs* safe/threat and threat/safe) (Table 3, task-consistency, and Figure 7). This effect is evident in the pooled data from the two tasks, highlighting a state-dependent, task-independent hippocampal effect. Importantly, the hippocampus ROI mask was fully encompassed by the final group EPI analysis mask.

## Discussion

We replicated our previous finding of impaired facial recognition performance (Bolton and Robinson, 2017) when anxiety was selectively induced at encoding. This behavioural effect appeared to be driven by greater ACC activation during encoding of faces (but not spatial stimuli) under threat relative to safe conditions. At the same time, we failed to replicate a behavioural state-dependent memory improvement in spatial span performance although, at the neural level, hippocampal activation appeared to reflect potential state-dependency at retrieval when the encoding state was reinstated. Interestingly, this state-dependent hippocampal effect was seen across both tasks.

The selective face recognition impairment during anxiety at encoding is consistent with the idea that anxiety may impair the formation of accurate face representations rather than acting on later recognition stages. Indeed, Attwood *et al.* (2013) showed anxiety induction disrupting face matching performance, where virtually no memory retention was required. At the neural level, increased ACC activation was observed during the anxiety induction at encoding. The ACC has, among other things, strongly been implicated in a so-called `attentional control network’, together with dlPFC and vlPFC (Duncan and Owen, 2000; MacDonald *et al.*, 2000), including attentional control over threat-related information (e.g. Bishop *et al.*, 2004; Ochsner and Gross, 2005). The dual competition model (Pessoa, 2009) proposes that ACC may help direct attention towards threat-related information, by receiving inputs about information salience from the amygdala. Therefore, in the present study, the ACC activation may reflect an increase in `top-down’ attentional resources allocated to anxiety processing. This change in attentional allocation may lead to a consequent reduction in the available top-down resources for forming accurate face representations at encoding (Brown *et al.*, 1997; Jackson and Raymond, 2006). Indeed, during learning, reductions in attentional allocation towards facial stimuli have previously been associated with reduced neural representations of faces (Pessoa *et al.*, 2002). Importantly, this ACC-related attentional mechanism may be selective to face processing, since it was not observed during the encoding of spatial configurations. Additionally, previous evidence showed increased ACC activation in phobic patients specifically during threat-related processing of facial stimuli (e.g. Amir *et al.*, 2005; Phan *et al.*, 2006). Therefore, in the present study, the ACC activation may indicate a selective mechanism by which threat disrupts the encoding of faces.

Furthermore, it is believed that the higher the state of threat, the more attentional control resources are engaged to process and prioritise threatening information (Pessoa, 2010). Consistent with this, the present study found that the degree of ACC activation was positively correlated with self-reported levels of stress. Thus, the more participants were affected by the anxiety manipulation, the more they allocated attention towards anxiety and the greater the ACC activation under anxiety. Although the ACC is implicated in a broad range of processes, the observed correlation between its activation and reported stress levels supports the selective involvement of the ACC in the encoding of faces during threat in the current experimental context.

The evidence that impaired face recognition under anxiety may arise from a reduction in attentional allocation towards goal-directed facial representations during encoding has important clinical implications as face recognition abnormalities have been implicated across anxiety disorders (e.g. Heuer *et al.*, 2007; Dickie *et al.*, 2008; Jarros *et al.*, 2012). Although face recognition impairments have previously been associated with reduced processing of facial stimuli (Mansell *et al.*, 1999; Chen *et al.*, 2002), the underlying neurocognitive mechanism was far from clear. The present study highlights a potential mechanism by which an anxiety-related reduction in top-down attentional resources at the level of the ACC selectively disrupts the encoding of face identities in the presence of anxiety.

Interestingly, however, the present study failed to replicate the spatial WM accuracy improvement when participants encoded and retrieved spatial information in the threat condition (Bolton and Robinson, 2017). At the neural level, greater hippocampus activation was recorded when both safe and threat conditions were matched across encoding and retrieval compared to when they mismatched, but this effect was seen across both tasks and was not evident in behavioural performance. Nevertheless, state-dependent hippocampal activation may be independent of the memory modality (i.e. domain general). Such involvement of the hippocampus in state-dependent effects would be consistent with its purported function of binding together information about context and target items (Montaldi and Mayes, 2010). Thus, hippocampal activity may correspond with the reactivation of associations between contextual information and target items (Diana *et al.*, 2007). As a result, associations between target spatial locations/faces and the emotional states (e.g. threat) in which they are learnt may be formed during encoding. Thus, when the same emotional state is reinstated, this reinstatement may reactivate the location/face–state associations, reflected by the hippocampal activation at retrieval observed in the present study (see Eich, 1989; Ucros, 1989; Lang *et al.*, 2001 and animal models: Rezayof *et al.*, 2007, 2008). Interestingly, the state-dependent activation was specifically observed in the posterior part of hippocampus. Unlike the anterior part (aHPC), posterior hippocampus (pHPC) activation has positively been related to the degree of context specificity and richness in environmental features with which past memory can be retrieved (Strange *et al.*, 2014; see Poppenk *et al.*, 2013 for a review on long-axis segregation of hippocampus). In the present study, it may be speculated that the state-dependent pHPC activity may reflect increased activation of contextual features associated to the encoded target items (i.e. facial and spatial stimuli), facilitated by the reinstatement of the same encoding state. This proposal would suggest that bodily sensations related to the encoding state may also be encoded as part of the richness of contextual information associated to specific experiences, explaining how state dependency may affect memory (e.g. see Eich, 1989; Ucros, 1989). In support to this view, mice studies have found that the dorsal hippocampus (i.e. equivalent to the pHPC in primates) responds to state-dependent memory effects (Rezayof *et al.*, 2007, 2008), thus supporting the specific involvement of the pHPC in state-dependent memory.

This state-dependent pHPC activation may thus contribute to the symptoms in PTSD and OCD patients, in which traumatic experiences or disturbing thoughts are repeatedly retrieved, especially as patients’ anxiety levels increase (Zlomuzica *et al.*, 2014).

Despite this hippocampal state-dependent activation, face recognition memory accuracy was unaffected at the behavioural level. In the literature, context-dependent memory effects have indeed rarely been found in relation to recognition paradigms (e.g. Godden and Baddeley, 1980; Smith *et al.*, 1978; Smith, 2013). This may be because recognition is supported by `familiarity processes’ (Gardiner and Java, 1990), which, unlike recollection (i.e. hippocampus dependent), may be unaffected by state/context-dependent memory (Macken, 2002). Consequently, the reliance of the face recognition task on familiarity processes may have prevented from recoding any observable memory improvement in recognition accuracy, despite item-state associations still triggering hippocampal activation.

However, it is not clear why spatial WM accuracy was unaffected, despite state-dependent hippocampal activation. Especially because a state-dependent memory improvement was observed in a previous study using the same task (i.e. Bolton and Robinson, 2017). It is possible that the combination of electrical shocks and the fMRI environment might have selected for a less anxious group, who were therefore resistant to the effect of TOS on spatial WM. This does not explain why the facial effect was preserved, however, so it is also possible that the original effect was a false positive.

In conclusion, we replicate the experiment by Bolton and Robinson (2017) finding that ToS selectively disrupts the encoding of facial stimuli and provide a putative neurocognitive mechanism. Specifically, increased threat-related processes may enhance competition for top-down attentional resources by engaging the ACC and impair the encoding of facial stimuli by increasing competition for top-down attentional resources hence diminishing the attentional pool necessarily to accurately encode facial stimuli. However, we failed to replicate spatial WM behavioural changes under induced anxiety, perhaps due to fMRI-related task or recruitment confounds. Nevertheless, a state-dependent task-irrelevant pHPC activation was observed when both encoding and retrieval occurred under the same condition, which may contribute to maladaptive retrieval processes observed in anxiety disorders.

## Funding

This work was supported by a Medical Research Council Career Development Award (MR/K024280/1) to OJR.

## Conflict of interest

The authors declare no competing financial interests.

## References

[ref1] Airaksinen E., Larsson M., Forsell Y. (2005). Neuropsychological functions in anxiety disorders in population-based samples: evidence of episodic memory dysfunction. Journal of Psychiatric Research, 39(2), 207–14. 15589570 10.1016/j.jpsychires.2004.06.001

[ref2] Amir N., Klumpp H., Elias J., Bedwell J.S., Yanasak N., Miller L.S. (2005). Increased activation of the anterior cingulate cortex during processing of disgust faces in individuals with social phobia. Biological Psychiatry, 57(9), 975–81. 15860337 10.1016/j.biopsych.2005.01.044

[ref3] Attwood A.S., Penton-Voak I.S., Burton A.M., Munafò M.R. (2013). Acute anxiety impairs accuracy in identifying photographed faces. Psychological Science, 24(8), 1591–4. 23780726 10.1177/0956797612474021

[ref4] Bishop S.J. (2009). Trait anxiety and impoverished prefrontal control of attention. Nature Neuroscience, 12, 92–8. 19079249 10.1038/nn.2242

[ref5] Bishop S., Duncan J., Brett M., Lawrence A.D. (2004). Prefrontal cortical function and anxiety: controlling attention to threat-related stimuli. Nature Neuroscience, 7(2), 184–8. 14703573 10.1038/nn1173

[ref6] Boldrini M., Del Pace L., Placidi G.P., et al. (2005). Selective cognitive deficits in obsessive-compulsive disorder compared to panic disorder with agoraphobia. Acta Psychiatrica Scandinavica, 111(2), 150–8. 15667435 10.1111/j.1600-0447.2004.00247.x

[ref7] Bolton S., Robinson O.J. (2017). The impact of threat of shock-induced anxiety on memory encoding and retrieval. Learning and Memory, 24(10), 432–42. 28916628 10.1101/lm.045187.117 PMC5602344

[ref8] Brown V., Huey D., Findlay J.M. (1997). Face detection in peripheral vision: do faces pop out? Perception, 26, 1555–70. 9616483 10.1068/p261555

[ref9] Chen Y.P., Ehlers A., Clark D.M., Mansell W. (2002). Patients with generalized social phobia direct their attention away from faces. Behaviour Research and Therapy, 40(6), 677–87. 12051486 10.1016/s0005-7967(01)00086-9

[ref10] Corbetta M., Shulman G.L. (2002). Control of goal-directed and stimulus-driven attention in the brain. Nature reviews. Neuroscience, 3(3), 201–15. 11994752 10.1038/nrn755

[ref11] Diana R.A., Yonelinas A.P., Ranganath C. (2007). Imaging recollection and familiarity in the medial temporal lobe: a three-component model. Trends in Cognitive Sciences, 11(9), 379–86. 17707683 10.1016/j.tics.2007.08.001

[ref12] Dickie E.W., Brunet A., Akerib V., Armony J.L. (2008). An fMRI investigation of memory encoding in PTSD: influence of symptom severity. Neuropsychologia, 46(5), 1522–31. 18321537 10.1016/j.neuropsychologia.2008.01.007

[ref13] Duncan J., Owen A.M. (2000). Common regions of the human frontal lobe recruited by diverse cognitive demands. Trends in Neurosciences, 23(10), 475–83. 11006464 10.1016/s0166-2236(00)01633-7

[ref14] Eich E. (1989). Theoretical issues in state dependent memory. Varieties of Memory and Consciousness: Essays in Honour of Endel Tulving, Psychology Press, New York, 331–54.

[ref15] Eichenbaum H., Sauvage M., Fortin N., Komorowski R., Lipton P. (2012). Towards a functional organization of episodic memory in the medial temporal lobe. Neuroscience & Biobehavioral Reviews, 36(7), 1597–608. 21810443 10.1016/j.neubiorev.2011.07.006 PMC3227798

[ref16] Eysenck M.W., Calvo M.G. (1992). Anxiety and performance: the processing efficiency theory. Cognition & Emotion, 6(6), 409–34.

[ref17] Eysenck M.W., Derakshan N., Santos R., Calvo M.G. (2007). Anxiety and cognitive performance: attentional control theory. Emotion, 7, 336–53. 17516812 10.1037/1528-3542.7.2.336

[ref18] Friston K.J., Holmes A.P., Worsley K.J. (1999). How many subjects constitute a study? NeuroImage, 10, 1–5. 10385576 10.1006/nimg.1999.0439

[ref19] Gardiner J.M., Java R.I. (1990). Recollective experience in word and nonword recognition. Memory & Cognition, 18, 23–30. 2314224 10.3758/bf03202642

[ref20] Godden D., Baddeley A. (1980). When does context influence recognition memory? British Journal of Psychology, 71, 99–104.

[ref21] Heuer K., Rinck M., Becker E.S. (2007). Avoidance of emotional facial expressions in social anxiety: the approach–avoidance task. Behaviour Research and Therapy, 45(12), 2990–3001. 17889827 10.1016/j.brat.2007.08.010

[ref22] Hopfinger J.B., Buonocore M.H., Mangun G.R. (2000). The neural mechanisms of top-down attentional control. Nature Neuroscience, 3(3), 284–91. 10700262 10.1038/72999

[ref23] Jackson M.C., Raymond J.E. (2006). The role of attention and familiarity in face identification. Attention, Perception, & Psychophysics, 68(4), 543–57. 16933420 10.3758/bf03208757

[ref24] Jarros R.B., Salum G.A., da Silva C.T.B., et al. (2012). Anxiety disorders in adolescence are associated with impaired facial expression recognition to negative valence. Journal of Psychiatric Research, 46(2), 147–51. 22018638 10.1016/j.jpsychires.2011.09.023

[ref25] Kanwisher N., McDermott J., Chun M.M. (1997). The fusiform face area: a module in human extrastriate cortex specialized for face perception. The Journal of Neuroscience, 17, 4302–11. 9151747 10.1523/JNEUROSCI.17-11-04302.1997 PMC6573547

[ref26] Kaye J.T., Bradford D.E., Curtin J.J. (2016). Psychometric properties of startle and corrugator response in NPU, affective picture viewing, and resting state tasks. Psychophysiology, 53(8), 1241–55. 27167717 10.1111/psyp.12663 PMC4949104

[ref27] Kessler R.C., Aguilar-Gaxiola S., Alonso J., et al. (2009). The global burden of mental disorders: an update from the WHO World Mental Health (WMH) surveys. Epidemiology and Psychiatric Sciences, 18, 23–33. 10.1017/s1121189x00001421 PMC3039289 19378696

[ref29] Kirlic N., Aupperle R.L., Rhudy J.L., et al. (2019). Latent variable analysis of negative affect and its contributions to neural responses during shock anticipation. Neuropsychopharmacology, 44(4), 695–702. 30181595 10.1038/s41386-018-0187-5 PMC6372706

[ref30] Lang A.J., Craske M.G., Brown M., Ghaneian A. (2001). Fear-related state dependent memory. Cognition & Emotion, 15(5), 695–703.

[ref31] Ma D.S., Correll J., Wittenbrink B. (2015). The Chicago face database: a free stimulus set of faces and norming data. Behavior Research Methods, 47, 1122–35. 25582810 10.3758/s13428-014-0532-5

[ref32] MacDonald A.W., Cohen J.D., Stenger V.A., Carter C.S. (2000). Dissociating the role of the dorsolateral prefrontal and anterior cingulate cortex in cognitive control. Science, 288, 1835–8. 10846167 10.1126/science.288.5472.1835

[ref33] Macken W.J. (2002). Environmental context and recognition: the role of recollection and familiarity. Journal of Experimental Psychology: Learning, Memory, and Cognition, 28, 153–61. 10.1037/0278-7393.28.1.153 11827077

[ref34] Mansell W., Clark D.M., Ehlers A., Chen Y.P. (1999). Social anxiety and attention away from emotional faces. Cognition & Emotion, 13(6), 673–90.

[ref35] Mantella R.C., Butters M.A., Dew M.A., et al. (2007). Cognitive impairment in late-life generalized anxiety disorder. The American Journal of Geriatric Psychiatry, 15(8), 673–9. 17426260 10.1097/JGP.0b013e31803111f2

[ref36] Martin C.B., Cowell R.A., Gribble P.L., Wright J., Köhler S. (2016). Distributed category-specific recognition-memory signals in human perirhinal cortex. Hippocampus, 26(4), 423–36. 26385759 10.1002/hipo.22531

[ref37] Montaldi D., Mayes A.R. (2010). The role of recollection and familiarity in the functional differentiation of the medial temporal lobes. Hippocampus, 20(11), 1291–314. 20928828 10.1002/hipo.20853

[ref38] Moran T.P. (2016). Anxiety and working memory capacity: a meta-analysis and narrative review. Psychological Bulletin, 142(8), 831–64. 26963369 10.1037/bul0000051

[ref39] Ochsner K.N., Gross J.J. (2005). The cognitive control of emotion. Trends in Cognitive Sciences, 9(5), 242–9. 15866151 10.1016/j.tics.2005.03.010

[ref40] Palermo R., Rhodes G. (2002). The influence of divided attention on holistic face perception. Cognition, 82(3), 225–57. 11747863 10.1016/s0010-0277(01)00160-3

[ref41] Palermo R., Rhodes G. (2007). Are you always on my mind? A review of how face perception and attention interact. Neuropsychologia, 45, 75–92. 16797607 10.1016/j.neuropsychologia.2006.04.025

[ref42] Pessoa L. (2009). How do emotion and motivation direct executive control? Trends in Cognitive Sciences, 13(4), 160–6. 19285913 10.1016/j.tics.2009.01.006 PMC2773442

[ref43] Pessoa L. (2010). Emergent processes in cognitive-emotional interactions. Dialogues in Clinical Neuroscience, 12(4), 433–88. 21319489 10.31887/DCNS.2010.12.4/lpessoa PMC3117594

[ref44] Pessoa L., McKenna M., Gutierrez E., Ungerleider L.G. (2002). Neural processing of emotional faces requires attention. Proceedings of the National Academy of Sciences, 99(17), 11458–63. 10.1073/pnas.172403899 PMC123278 12177449

[ref45] Phan K.L., Fitzgerald D.A., Nathan P.J., Tancer M.E. (2006). Association between amygdala hyperactivity to harsh faces and severity of social anxiety in generalized social phobia. Biological Psychiatry, 59(5), 424–9. 16256956 10.1016/j.biopsych.2005.08.012

[ref46] Poppenk J., Evensmoen H.R., Moscovitch M., Nadel L. (2013). Long-axis specialization of the human hippocampus. Trends in Cognitive Sciences, 17(5), 230–40. 23597720 10.1016/j.tics.2013.03.005

[ref47] Rezayof A., Motevasseli T., Rassouli Y., Zarrindast M.R. (2007). Dorsal hippocampal dopamine receptors are involved in mediating ethanol state-dependent memory. Life Sciences, 80(4), 285–92. 17046026 10.1016/j.lfs.2006.09.013

[ref48] Rezayof A., Alijanpour S., Zarrindast M.R., Rassouli Y. (2008). Ethanol state-dependent memory: involvement of dorsal hippocampal muscarinic and nicotinic receptors. Neurobiology of Learning and Memory, 89(4), 441–7. 18065245 10.1016/j.nlm.2007.10.011

[ref49] Robinson O.J., Charney D.R., Overstreet C., Vytal K., Grillon C. (2012). The adaptive threat bias in anxiety: amygdala–dorsomedial prefrontal cortex coupling and aversive amplification. NeuroImage, 60, 523–9. 22178453 10.1016/j.neuroimage.2011.11.096 PMC3288162

[ref50] Robinson O.J., Vytal K., Cornwell B.R., Grillon C. (2013). The impact of anxiety upon cognition: perspectives from human threat of shock studies. Frontiers in Human Neuroscience, 7, 203. 23730279 10.3389/fnhum.2013.00203 PMC3656338

[ref51] Robinson O.J., Krimsky M., Lieberman L., Vytal K., Ernst M., Grillon C. (2016). Anxiety-potentiated amygdala–medial frontal coupling and attentional control. Translational Psychiatry, 6(6), e833. 27271859 10.1038/tp.2016.105 PMC4931603

[ref52] Shackman A.J., Sarinopoulos I., Maxwell J.S., Pizzagalli D.A., Lavric A., Davidson R.J. (2006). Anxiety selectively disrupts visuospatial working memory. Emotion, 6(1), 40–61. 16637749 10.1037/1528-3542.6.1.40

[ref53] Smith S.M., Glenberg A., Bjork R.A. (1978). Environmental context and human memory. Memory & Cognition, 6(4), 342–353.

[ref54] Smith S.M. (2013). Effects of environmental context on human memory. The SAGE Handbook of Applied Memory, Sage, London, 162–82.

[ref55] Strange B.A., Witter M.P., Lein E.S., Moser E.I. (2014). Functional organization of the hippocampal longitudinal axis. Nature Reviews Neuroscience, 15(10), 655–69. 25234264 10.1038/nrn3785

[ref56] Straube T., Mentzel H.J., Miltner W.H. (2007). Waiting for spiders: brain activation during anticipatory anxiety in spider phobics. NeuroImage, 37(4), 1427–36. 17681799 10.1016/j.neuroimage.2007.06.023

[ref57] Straube T., Schmidt S., Weiss T., Mentzel H.J., Miltner W.H. (2009). Dynamic activation of the anterior cingulate cortex during anticipatory anxiety. NeuroImage, 44(3), 975–81. 19027072 10.1016/j.neuroimage.2008.10.022

[ref58] Surcinelli P., Codispoti M., Montebarocci O., Rossi N., Baldaro B. (2006). Facial emotion recognition in trait anxiety. Journal of Anxiety Disorders, 20, 110–7. 16325118 10.1016/j.janxdis.2004.11.010

[ref59] Tzourio-Mazoyer N., Landeau B., Papathanassiou D., et al. (2002). Automated anatomical labeling of activations in SPM using a macroscopic anatomical parcellation of the MNI MRI single-subject brain. NeuroImage, 15, 273–89. 11771995 10.1006/nimg.2001.0978

[ref60] Ucros C.G. (1989). Mood state-dependent memory: a meta-analysis. Cognition and Emotion, 3, 139–69.

[ref61] Vytal K., Cornwell B., Arkin N., Grillon C. (2012). Describing the interplay between anxiety and cognition: from impaired performance under low cognitive load to reduced anxiety under high load. Psychophysiology, 49, 842–52. 22332819 10.1111/j.1469-8986.2012.01358.x PMC3345059

[ref62] Vytal K.E., Cornwell B.R., Letkiewicz A.M., Arkin N.E., Grillon C. (2013). The complex interaction between anxiety and cognition: insight from spatial and verbal working memory. Frontiers in Human Neuroscience, 7, 93. 23542914 10.3389/fnhum.2013.00093 PMC3610083

[ref63] Wee N.J. van der, Ramsey N.F., Jansma J.M., et al. (2003). Spatial working memory deficits in obsessive compulsive disorder are associated with excessive engagement of the medial frontal cortex. NeuroImage, 20, 2271–80. 14683728 10.1016/j.neuroimage.2003.05.001

[ref64] Woo C.W., Krishnan A., Wager T.D. (2014). Cluster-extent based thresholding in fMRI analyses: pitfalls and recommendations. NeuroImage, 91, 412–9. 24412399 10.1016/j.neuroimage.2013.12.058 PMC4214144

[ref65] Zlomuzica A., Dere D., Machulska A., Adolph D., Dere E., Margraf J. (2014). Episodic memories in anxiety disorders: clinical implications. Frontiers in Behavioral Neuroscience, 8, 131. 24795583 10.3389/fnbeh.2014.00131 PMC4005957

